# Construction of a Mortality Prediction Model for Cardiac Arrest Patients Using Lactate Clearance Rate and Albumin‐Corrected Anion Gap

**DOI:** 10.1155/emmi/3964486

**Published:** 2026-08-17

**Authors:** Yani Gao, Sheng Bi, Haoran Li, Xiaojin Sun, Chaogang Wei, Xuming Zhao, Yan Xiao

**Affiliations:** ^1^ Department of Critical Care Medicine, Sihong Hospital, Suqian, Jiangsu, China; ^2^ Department of Emergency and Critical Care Medicine, The Second Affiliated Hospital of Soochow University, Suzhou, Jiangsu, China, suda.edu.cn; ^3^ Department of Cardiovascular Medicine, Sihong Hospital, Suqian, Jiangsu, China; ^4^ Department of Radiology, The Second Affiliated Hospital of Soochow University, Suzhou, Jiangsu, China, suda.edu.cn; ^5^ Department of Critical Care and Rehabilitation Medicine, Shanghai Hebin Rehabilitation Hospital, Shanghai, China; ^6^ Department of Emergency Medicine, Putuo Hospital, Shanghai University of Traditional Chinese Medicine, Shanghai, China, shutcm.edu.cn

**Keywords:** albumin-corrected anion gap, cardiac arrest, lactic clearance rate, MIMIC database, predictive model

## Abstract

**Background:**

Cardiac arrest (CA) causes systemic circulatory failure, leading to postcardiac arrest syndrome (PCAS) and high mortality. Lactic clearance rate (LCR) and albumin‐corrected anion gap (ACAG) reflect metabolic status but lack combined validation for predicting short‐term mortality in CA patients.

**Objective:**

This study explores the predictive value of LCR at different time points and ACAG for 7‐day mortality in post‐CA patients and constructs a predictive model.

**Methods:**

Using the MIMIC‐IV 3.0 database, we included eligible CA patients and calculated LCR at 6, 12, and 24 h, along with initial ACAG. ROC curves and logistic regression were used to assess predictive value, while restricted cubic splines (RCS) explored mortality risk. The best predictors were selected for model construction, with multivariate logistic regression identifying independent risk factors. Internal and external validations were performed.

**Results:**

A total of 821 patients were included, with a 7‐day mortality rate of 34.47%. LCR was lower, and ACAG was higher in nonsurvivors (*p* < 0.05). The 24‐h LCR combined with ACAG had the highest predictive efficacy (area under the ROC curve [AUC] = 0.716). Independent risk factors included 24‐h LCR, ACAG, CK–MB, shockable rhythm, and CA etiology. The final model achieved an AUC of 0.775 (internal) and 0.743 (external).

**Conclusion:**

The 24‐h LCR combined with ACAG predicts short‐term mortality in post‐CA patients who survive the first 24 h, supporting risk stratification after early postresuscitation stabilization.

## 1. Introduction

Cardiac arrest (CA) is a condition where the heart suddenly stops pumping blood, leading to the cessation of systemic circulation, resulting in respiratory arrest and loss of consciousness [1]. Despite the critical role of cardiopulmonary resuscitation (CPR) and advanced life support (ALS) in emergency care, the survival rate of patients after resuscitation remains low [2]. Early postresuscitation mortality is primarily caused by postcardiac arrest syndrome (PCAS) [3], which includes brain injury, myocardial dysfunction, systemic ischemia–reperfusion response, and persistent pathophysiological reactions [4]. Accurately assessing the short‐term mortality risk of patients to optimize intervention measures remains a hot and challenging topic in clinical research.

Lactate is increasingly viewed as a marker of systemic metabolic stress and energy‐state dysregulation rather than a direct measure of tissue perfusion or oxygenation alone. It has been widely used in the prognosis assessment of critically ill patients and is a recognized prognostic marker for sepsis and cardiogenic shock [5, 6]. Lactate clearance rate (LCR) dynamically assesses the metabolic state of patients, and recent studies have revealed a significant association between LCR and patient mortality [7, 8]. A cohort study of 64 CA patients suggested that LCR is one of the most important factors in predicting mortality [9].

Acid‐base imbalance, especially metabolic acidosis, which is common in ICU patients, has been proven to be associated with the morbidity and mortality of various diseases [10]. Anion gap (AG) is one of the most commonly used biomarkers for assessing acid‐base imbalance, representing the difference between unmeasured cations and unmeasured anions in serum. However, albumin constitutes a large portion of unmeasured anions, changes in its concentration can significantly affect the calculation of the AG. In patients with hypoalbuminemia, uncorrected AG may underestimate the severity of metabolic acidosis [11]. The albumin‐corrected anion gap (ACAG) adjusts the original AG value based on albumin levels to improve its accuracy in assessing metabolic acid‐base imbalance [12]. A previous study demonstrated that ACAG is a significant prognostic marker in patients with cardiopulmonary arrest, supporting its utility in this population [13]. Recent evidence also supports lactate clearance as a prognostic tool in CPR‐related outcomes and very early mortality endpoints [14].

LCR and ACAG capture related but distinct aspects of post‐arrest metabolic derangement. However, evidence evaluating their combined use in a single prediction model for short‐term mortality after CA remains limited. This study, based on the MIMIC‐IV database, aims to explore the predictive value of LCR and ACAG for short‐term mortality in CA patients and to construct a mortality risk prediction model to evaluate its clinical applicability.

## 2. Methods

### 2.1. Data Source

**FIGURE 1:**
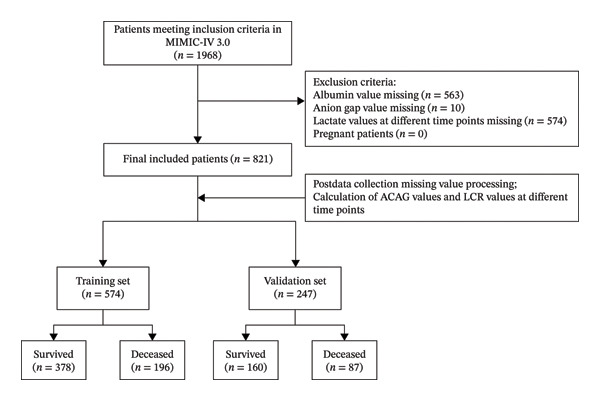
MIMIC database data extraction flowchart.

**FIGURE 2:**
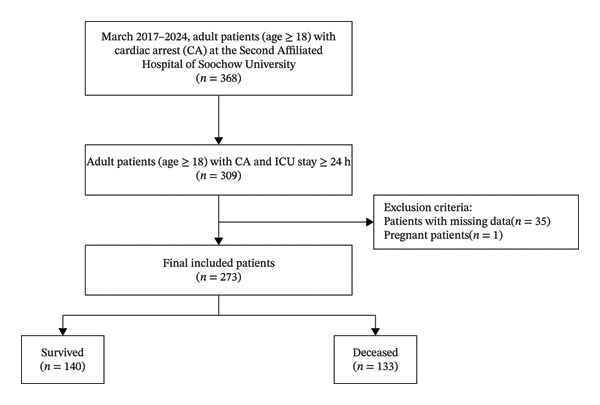
The Second Affiliated Hospital of Soochow University data extraction flowchart.

This study extracted adult patients (≥ 18 years) diagnosed with CA between 2008 and 2022 from the MIMIC‐IV 3.0 database based on International Classification of Diseases (ICD‐9 and ICD‐10) diagnostic codes (“4275,” “I46,” “I462,” “I468,” “I469,” “I97710,” “I97711,” “I97120,” “I97121”). Due to the structure of MIMIC‐IV and the ICD‐code–based extraction strategy, the exact location of CA could not be reliably determined for all patients; therefore, the derivation cohort should be interpreted as a mixed post‐CA population rather than a cohort specifically separated into out‐of‐hospital and in‐hospital CA. A total of 1968 patients meeting the criteria of ROSC ≥ 24 h and age ≥ 18 years were initially included. After excluding 1147 cases (58%) with missing data, the final cohort comprised 821 patients (Figure 1). Additionally, data from CA patients meeting the inclusion criteria from the electronic medical record system of the Second Affiliated Hospital of Soochow University from March 2017 to August 2024 were collected (Figure 2). Exclusion criteria included: (1) missing key laboratory test results (AG, albumin, lactate values at different time points) and (2) pregnancy. All clinical data were extracted from the first 24 h of ICU admission, and vital signs and laboratory tests were taken from the first measurement within 24 h.

The target population of this study comprised post‐CA patients who achieved ROSC and survived at least 24 h with serial lactate, AG, and albumin measurements available. Accordingly, the model was designed to support risk stratification among patients who had survived the initial 24‐h postresuscitation period rather than to guide immediate therapeutic decisions during the first hours after ROSC. This inclusion criterion may introduce survival/availability bias by excluding early deaths and patients without repeated laboratory sampling.

MIMIC is a comprehensive, one‐stop, publicly accessible database containing patient data from the intensive care units of a large tertiary medical institution [15, 16]. Since 2008, the database has recorded data from over 90,000 patients. Before accessing the database, the authors completed the Collaborative Institutional Training Initiative (CITI) course and passed the ethics exam, obtaining a CITI certificate (certificate number 62057797), thus being approved to extract data from the database.

This study has also been approved by the Ethics Review Committee of the Second Affiliated Hospital of Soochow University (ethics number: JD‐HG‐2024‐102).

### 2.2. Outcomes and Definitions

The primary outcome was 7‐day all‐cause mortality after ICU admission (7‐day ICU mortality). Patients discharged alive from the ICU before Day 7 were considered alive at Day 7 unless death was recorded in the hospital database within 7 days of admission. Cause‐specific death was not analyzed because MIMIC‐IV does not provide a reliable adjudicated field distinguishing cardiac death, neurological death, or death due to multiorgan failure. In addition, formal neurological injury or neurological outcome variables were not consistently extractable from the database.

LCR, AG, and ACAG were calculated using standard formulas [17–19], which are provided in the Supporting Information. Negative LCR values, indicating a rise in lactate, were permitted and included in the analyses. Time zero was defined as ICU admission, which was used as a proxy for ROSC time due to database constraints. Baseline lactate was defined as the first lactate measurement obtained within 1 h after ICU admission. The 6‐, 12‐, and 24‐h lactate values were defined as the closest measurements to 6 ± 1, 12 ± 1, and 24 ± 1 h after ICU admission, respectively. If multiple measurements existed within the window, the one closest to the target time was selected; if no measurement was available, the value was treated as missing.

A shockable rhythm was defined as the initial recorded rhythm being ventricular fibrillation (VF) or pulseless ventricular tachycardia (pVT), extracted from the resuscitation event documentation.

### 2.3. Statistical Analysis

Continuous variables were summarized as mean ± standard deviation or median (interquartile range) and categorical variables as number (percentage). Between‐group comparisons used the *t*‐test or Wilcoxon rank‐sum test for continuous variables and the chi‐square test or Fisher’s exact test for categorical variables, as appropriate. Samples missing lactate, AG, or albumin data required for LCR/ACAG calculation were excluded; variables with > 30% missingness were not included, and remaining covariate missingness was handled using multiple imputation with chained equations (5 imputations; details in Supporting Information).

The MIMIC‐IV cohort was randomly divided into a training set and an internal validation set in a 7:3 ratio, and the Second Affiliated Hospital of Soochow University cohort was used for external validation. Candidate predictors associated with 7‐day all‐cause mortality were evaluated using logistic regression. Multicollinearity was assessed using the variance inflation factor (VIF), with VIF < 10 indicating no substantial multicollinearity. Model discrimination was assessed using receiver operating characteristic (ROC) curves and area under the ROC curve (AUC); calibration was assessed using calibration curves, the Hosmer–Lemeshow (H–L) test, and Brier score; and clinical utility was assessed using decision curve analysis. A complete‐case sensitivity analysis was performed to evaluate the robustness of the imputation‐based results.

Restricted cubic splines (RCS) were used only as an exploratory logistic‐regression–based analysis to visualize possible nonlinear associations between LCR/ACAG and binary 7‐day mortality. They were not used as a time‐to‐event survival model or for predictor selection. Statistical analyses were performed using RStudio 4.4.1, SPSS 27.0.1, Zstats, and GraphPad Prism 10.1.2, with *p* < 0.05 considered statistically significant.

## 3. Results

### 3.1. Basic Information

According to the inclusion and exclusion criteria, data from 821 eligible patients were extracted from the MIMIC‐IV database, including 283 deaths and 538 survivors. Variables with missing values > 30% were deleted, as shown in Table S1, and missing values for other variables were imputed using multiple imputations in RStudio 4.4.1.

### 3.2. Baseline Characteristics of Patients in the MIMIC‐IV Database

The baseline characteristics of patients in the MIMIC‐IV database showed significant differences between the two groups in 6‐h LCR, 12‐h LCR, 24‐h LCR, ACAG, white blood cell count, red blood cell count, platelet count, hemoglobin, phosphate, hematocrit, blood glucose, pH, partial pressure of oxygen, prothrombin time, International Normalized Ratio, alanine aminotransferase, aspartate aminotransferase, serum creatinine, creatine kinase, creatine kinase–MB, troponin T, use of norepinephrine, shockable rhythm, cardiac origin of arrest, and SOFA score (all *p* < 0.05). Details are shown in Table 1.

**TABLE 1:** Baseline characteristics of cardiac arrest patients with ROSC admitted to the ICU.

Parameter	Total (after imputation)	Total (before imputation)	Missing rate (%)	Survivor group	Nonsurvivor group	*χ* ^2^/*Z*	*p*
	(*n* = 821)	(*n* = 821)		(*n* = 538)	(*n* = 283)		
Age (years)	66 (55, 77)	66 (55, 77)	0	66 (56, 78)	66 (54, 77)	*Z* = −0.52	0.604
Weight (kg)	81.8 (69.5, 98.4)	81.6 (69.4, 98.4)	1.8	81 (68.9, 97.2)	84 (70.2, 100.1)	*Z* = −1.12	0.263
Height (cm)	170 (163, 178)	170 (163, 178)	22.9	170 (163, 178)	173 (163, 178)	*Z* = −0.42	0.674
Gender							
Male, *n* (%)	519 (63.2)	519 (63.2)	0	347 (64.5)	172 (60.8)	*χ* ^2^ = 1.10	0.293
Female, *n* (%)	302 (36.8)	302 (36.8)	0	191 (35.5)	111 (39.2)		
Complication							
Hypertension, *n* (%)	278 (33.9)	278 (33.9)	0	184 (34.2)	94 (33.2)	*χ* ^2^ = 0.08	0.777
Diabetes, *n* (%)	286 (34.8)	286 (34.8)	0	193 (35.9)	93 (32.9)	*χ* ^2^ = 0.74	0.389
Myocardial infarction, *n* (%)	170 (20.7)	170 (20.7)	0	108 (20.1)	62 (21.9)	*χ* ^2^ = 0.38	0.538
Renal dysfunction, *n* (%)	575 (70.0)	575 (70.0)	0	370 (68.8)	205 (72.4)	*χ* ^2^ = 1.19	0.276
Stroke, *n* (%)	77 (9.4)	77 (9.4)	0	53 (9.9)	24 (8.5)	*χ* ^2^ = 0.41	0.522
Chronic liver disease, *n* (%)	55 (6.7)	55 (6.7)	0	37 (6.9)	18 (6.4)	*χ* ^2^ = 0.08	0.778
Malignancy, *n* (%)	83 (10.1)	83 (10.1)	0	47 (8.7)	36 (12.7)	*χ* ^2^ = 3.24	0.072
Sepsis, *n* (%)	268 (32.6)	268 (32.6)	0	183 (34.1)	85 (30.1)	*χ* ^2^ = 1.34	0.248
6‐h LCR (%)	30.19 (4.62, 54.41)	30.19 (4.62, 54.41)	0	36.36 (8.56, 58.22)	20.78 (0.00, 43.58)	*Z* = −4.90	< 0.001
12‐h LCR (%)	50.94 (25.00, 68.83)	50.94 (25.00, 68.83)	0	55.14 (30.80, 72.31)	40.00 (12.11, 63.11)	*Z* = −5.95	< 0.001
24‐h LCR (%)	63.16 (38.46, 76.92)	63.16 (38.46, 76.92)	0	66.67 (46.60, 79.75)	53.23 (13.39, 69.95)	*Z* = −7.29	< 0.001
ACAG (mmol/L)	21.0 (17.5, 24.8)	21.0 (17.5, 24.8)	0	19.8 (16.8, 23.9)	22.5 (19.0, 27.1)	*Z* = −5.88	< 0.001
WBC (10^9/L)	14.2 (9.3, 20.0)	14.2 (9.3, 20.0)	0.1	13.9 (9.1, 19.3)	15.0 (9.7, 22.0)	*Z* = −2.21	0.027
RBC (10^12/L)	3.66 (3.00, 4.43)	3.66 (3.00, 4.43)	0.1	3.58 (2.91, 4.40)	3.79 (3.13, 4.55)	*Z* = −2.48	0.013
PLT (10^9/L)	193 (135, 260)	193 (137, 261)	0.4	187 (134, 242)	208 (143, 286)	*Z* = −2.87	0.004
HB (g/dL)	10.9 (8.9, 13.0)	10.9 (8.9, 13.0)	0.1	10.7 (8.7, 12.9)	11.3 (9.3, 13.6)	*Z* = −2.82	0.005
PO_4_^3−^ (mg/dL)	4.40 (3.20, 5.80)	4.40 (3.20, 5.82)	0.1	4.20 (3.10, 5.60)	4.70 (3.45, 6.40)	*Z* = −3.37	< 0.001
RDW (%)	14.7 (13.6, 16.4)	14.7 (13.6, 16.4)	0.1	14.7 (13.5, 16.4)	14.7 (13.6, 16.6)	*Z* = −1.33	0.184
HCT (%)	34.0 (28.1, 40.8)	34.0 (28.1, 40.8)	0.4	33.1 (27.3, 40.0)	35.1 (29.1, 42.0)	*Z* = −3.02	0.003
Na^+l^ (mmol/L)	139 (135, 142)	139 (135, 142)	0	139 (135, 142)	140 (136, 142)	*Z* = −0.99	0.325
K^+^ (mmol/L)	4.30 (3.80, 4.90)	4.30 (3.80, 4.90)	0.2	4.30 (3.80, 4.90)	4.30 (3.70, 4.90)	*Z* = −0.18	0.861
Cl^−^ (mmol/L)	104 (99, 108)	104 (99, 108)	0	105 (100, 109)	104 (99, 108)	*Z* = −1.86	0.063
Ca^2+^ (mmol/L)	1.10 (1.03, 1.17)	1.10 (1.03, 1.17)	7.7	1.10 (1.04, 1.17)	1.09 (1.03, 1.17)	*Z* = −1.53	0.126
GLU (mg/dL)	177 (129, 253)	177 (129, 253)	0	168 (123, 242)	196 (140, 264)	*Z* = −3.01	0.003
PH	7.28 (7.18, 7.36)	7.28 (7.18, 7.36)	0	7.30 (7.20, 7.38)	7.24 (7.16, 7.34)	*Z* = −4.25	< 0.001
PCO_2_ (mmHg)	43.0 (36.0, 52.0)	43.0 (36.0, 52.0)	0.1	43.0 (36.0, 51.0)	43.0 (36.5, 52.0)	*Z* = −0.57	0.571
PO_2_ (mmHg)	91.0 (50.0.200.5)	91.0 (50.0, 200.5)	0.2	103.5 (50.0, 218.8)	81.0 (46.0, 152.0)	*Z* = −3.18	0.001
PT (s)	15.10 (12.90, 19.70)	15.20 (13.00, 19.70)	2.2	14.90 (12.80, 18.70)	15.40 (13.30, 21.50)	*Z* = −2.35	0.019
INR	1.40 (1.20, 1.80)	1.40 (1.20, 1.80)	2.3	1.40 (1.20, 1.70)	1.40 (1.20, 2.00)	*Z* = −2.57	0.01
TBIL (mg/dL)	0.60 (0.40, 1.20)	0.60 (0.40, 1.20)	3.3	0.60 (0.40, 1.20)	0.60 (0.40, 1.15)	*Z* = −0.15	0.881
ALT (U/L)	71 0.00 (29.00, 209.00)	72.00 (29.00, 208.75)	3.3	57.00 (26.00, 152.00)	104.00 (42.00, 332.00)	*Z* = −5.24	< 0.001
AST (U/L)	113.00 (48.00, 347.00)	113.00 (48.00, 347.00)	2.6	87.00 (41.00, 245.00)	184.00 (81.00, 553.00)	*Z* = −7.14	< 0.001
BUN (mg/dL)	24 (17, 39)	24 (17, 39)	0	24 (16, 38)	25 (18, 42)	*Z* = −1.60	0.11
Cr (mg/dL)	1.40 (1.00, 2.20)	1.35 (1.00, 2.20)	0.1	1.30 (0.90, 2.10)	1.50 (1.10, 2.30)	*Z* = −3.08	0.002
CK (U/L)	346.0 (124.0, 1041.0)	328.5 (118.0, 1045.0)	25.5	274.0 (105.0, 829.0)	518.0 (186.0, 1722.0)	*Z* = −5.36	< 0.001
CK–MB (ng/mL)	9.00 (4.00, 28.00)	10.00 (4.00, 28.00)	23.6	8.00 (4.00, 19.00)	15.00 (6.00, 46.00)	*Z* = −6.86	< 0.001
cTnT (ng/mL)	0.22 (0.07, 0.75)	0.24 (0.08, 0.79)	27.9	0.20 (0.07, 0.68)	0.26 (0.09, 0.93)	*Z* = −2.20	0.028
Cardiogenic, *n* (%)	104 (12.67)	104 (12.67)	0	78 (14.50)	26 (9.19)	*χ* ^2^ = 4.73	0.03
Emergency admission, *n* (%)	136 (16.57)	136 (16.57)	0	94 (17.47)	42 (14.84)	*χ* ^2^ = 0.93	0.335
Shockable rhythm, *n* (%)	53 (6.46)	53 (6.46)	0	48 (8.92)	5 (1.77)	*χ* ^2^ = 15.72	< 0.001
Vasoactive agent							
Dopamine, *n* (%)	94 (11.45)	94 (11.45)	0	58 (10.78)	36 (12.72)	*χ* ^2^ = 0.69	0.407
Epinephrine, *n* (%)	185 (22.53)	185 (22.53)	0	115 (21.38)	70 (24.73)	*χ* ^2^ = 1.20	0.274
Norepinephrine, *n* (%)	514 (62.61)	514 (62.61)	0	317 (58.92)	197 (69.61)	*χ* ^2^ = 9.05	0.003
Renal replacement therapy, *n* (%)	162 (19.73)	162 (19.73)	0	97 (18.03)	65 (22.97)	*χ* ^2^ = 2.86	0.091
Invasive mechanical ventilation, *n* (%)	626 (76.25)	626 (76.25)	0	400 (74.35)	226 (79.86)	*χ* ^2^ = 3.11	0.078
Heart rate (bpm)	90 (77, 108)	90 (77, 108)	0	90 (76, 108)	90 (79, 108)	*Z* = −0.60	0.546
Systolic BP (mmHg)	115 (99, 132)	115 (99, 132)	23.5	115 (100, 131)	114 (97, 136)	*Z* = −0.72	0.472
Diastolic BP (mmHg)	62 (52, 73)	62 (52, 73)	23.5	61 (52, 73)	63 (52, 75)	*Z* = −0.95	0.342
MAP (mmHg)	79 (66, 93)	79 (66, 93)	23.3	78 (67, 91)	81 (65, 97)	*Z* = −1.04	0.298
Respiratory rate (bpm)	20 (16, 25)	20 (16, 25)	0	20 (16, 25)	20 (16, 25)	*Z* = −0.97	0.333
Oxygen saturation (%)	99 (95, 100)	99 (95, 100)	0	99 (95, 100)	99 (95, 100)	*Z* = −0.85	0.396
Temperature (°C)	36.8 (36.4, 37.2)	36.8 (36.4, 37.2)	8.4	36.8 (36.4, 37.2)	36.8 (36.4, 37.1)	*Z* = −0.71	0.48
SOFA score	9 (7, 12)	9 (7, 12)	0	9 (6, 11)	10 (7, 12)	*Z* = −4.46	< 0.001
SAPS II score	50.0 (39.0, 61.0)	50.0 (39.0, 61.0)	0	49.5 (38.0, 61.0)	50.0 (40.0, 59.5)	*Z* = −0.52	0.603
GCS score	15.0 (14.0, 15.0)	15.0 (14.0, 15.0)	0.1	15.0 (14.0, 15.0)	15.0 (13.0, 15.0)	*Z* = −1.15	0.248

*Note:* HB: hemoglobin; PO_4_^3−^: phosphate; HCT: hematocrit; Na^+^: sodium ion; K^+^: potassium ion; Cl^−^: chloride ion; Ca^2+^: calcium ion; GLU: blood glucose; pH: acidity/alkalinity level; PCO_2_: arterial partial pressure of carbon dioxide; PO_2_: arterial partial pressure of oxygen; Cr: creatinine; PTL: platelet count; TBIL: total bilirubin.

Abbreviations: 6‐h LCR, 6‐h lactate clearance rate; 12‐h LCR, 12‐h lactate clearance rate; 24‐h LCR, 24‐h lactate clearance rate; ACAG, albumin‐corrected anion gap; ALT, alanine aminotransferase; AST, aspartate aminotransferase; BUN, blood urea nitrogen; CK, creatine kinase; CK–MB, creatine kinase–MB isoenzyme; cTnT, cardiac troponin T; INR, international normalized ratio; PT, prothrombin time; RBC, red blood cell count; RDW, red cell distribution width; WBC, white blood cell count.

### 3.3. Analysis of the Predictive Value of Different Indicators or Methods for Patient Mortality in the MIMIC‐IV Database

#### 3.3.1. Univariate Logistic Analysis

Baseline characteristics showed that ACAG and LCR at different time points had significant differences. These indicators were included in univariate logistic regression analysis, and the results showed that ACAG and LCR at different time points were all influencing factors for patient mortality (*p* < 0.05), as shown in Table 2.

**TABLE 2:** Influencing factors of patient mortality in univariate logistic regression analysis.

Parameter	OR	95% CI	*p*
6‐h LCR	0.994	0.991–0.997	< 0.001
12‐h LCR	0.991	0.987–0.994	< 0.001
24‐h LCR	0.986	0.982–0.990	< 0.001
ACAG	1.067	1.042–1.093	< 0.001

Abbreviations: 6‐h LCR, 6‐h lactate clearance rate; 12‐h LCR, 12‐h lactate clearance rate; 24‐h LCR, 24‐h lactate clearance rate; ACAG, albumin‐corrected anion gap; CI, confidence interval; OR, odds ratio.

#### 3.3.2. Exploratory RCS Analysis

**FIGURE 3:**
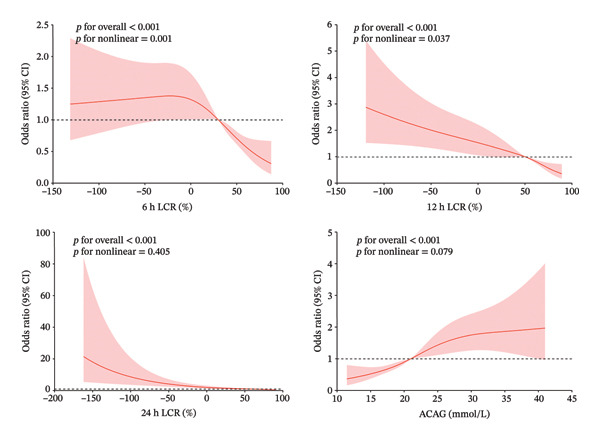
RCS trend graphs for LCR and ACAG at different time points.

Exploratory RSC analyses were performed to visualize possible nonlinear associations between LCR/ACAG and binary 7‐day mortality, not to model time‐to‐event survival. The curves suggested that 6 h LCR and 12 h LCR had nonlinear associations with 7‐day mortality risk (*p* for nonlinearity < 0.05), while 24‐h LCR and ACAG showed approximately linear associations (*p* for nonlinearity > 0.05). As LCR increased, 7‐day mortality risk gradually decreased; as ACAG increased, 7‐day mortality risk showed an upward trend (Figure 3). These exploratory findings were not used for predictor selection in the final model.

#### 3.3.3. ROC Curve Analysis

**FIGURE 4:**
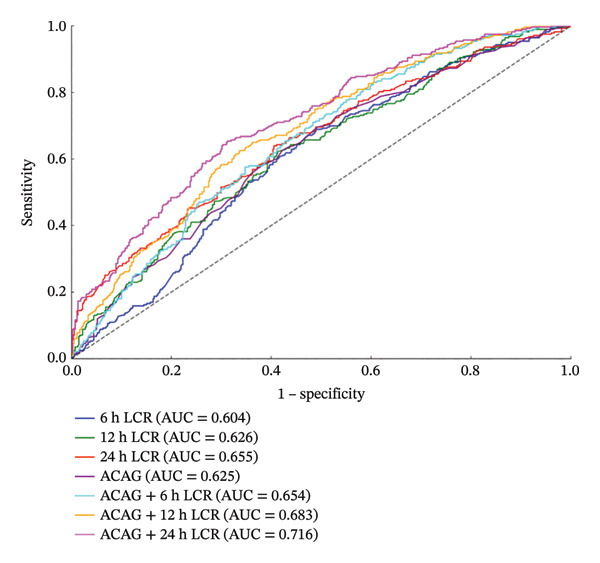
ROC curves for different indicators or methods predicting patient mortality.

The cutoff value corresponding to the maximum Youden index for each indicator predicting patient mortality was recorded. ACAG and LCR at different time points could all predict patient mortality, and the combination of 24‐h LCR and ACAG had a higher AUC than other time points combined with ACAG and also higher than each indicator alone. Results are shown in Table 3 and Figure 4. Therefore, 24‐h LCR and ACAG were used as the basis for constructing the mortality risk prediction model.

**TABLE 3:** Predictive value analysis of different indicators or methods for patient mortality.

Parameter	Cutoff value	AUC	95% CI	*p*
6‐h LCR	34.4%	0.604	0.564–0.644	< 0.001
12‐h LCR	49.8%	0.626	0.586–0.666	< 0.001
24‐h LCR	62.2%	0.655	0.615–0.694	< 0.001
ACAG	19.88	0.625	0.585–0.665	< 0.001
ACAG + 6‐h LCR	—	0.654	0.616–0.692	< 0.001
ACAG + 12‐h LCR	—	0.683	0.646–0.720	< 0.001
ACAG + 24‐h LCR	—	0.716	0.680–0.752	< 0.001

*Note:* AUC, area under the receiver operating characteristic curve.

Abbreviations: 6‐h LCR, 6‐h lactate clearance rate; 12‐h LCR, 12‐h lactate clearance rate; 24‐h LCR, 24‐h lactate clearance rate; ACAG, albumin‐corrected anion gap; CI, confidence interval.

### 3.4. A Mortality Risk Prediction Model Was Constructed Based on 24‐h LCR and ACAG

#### 3.4.1. Univariate Logistic Regression Analysis in the MIMIC‐IV Database

Univariate logistic regression analysis showed significant differences between the death and survival groups in 24‐h LCR, ACAG, white blood cell count, red blood cell count, platelet count, hemoglobin, phosphate, hematocrit, blood glucose, pH, partial pressure of oxygen, prothrombin time, alanine aminotransferase, aspartate aminotransferase, creatine kinase–MB, use of norepinephrine, SOFA score, cardiac origin of arrest, and shockable rhythm (all *p* < 0.05), while other indicators showed no significant differences (all *p* > 0.05). Results are shown in Table S2.

#### 3.4.2. Multivariate Logistic Regression Analysis in the MIMIC‐IV Database

Based on the results in Table S2, with 7‐day mortality in the ICU as the dependent variable and the 19 statistically significant variables as independent variables, binary logistic regression with the forward‐LR conditional method was used for regression analysis. The results showed that the 24‐h LCR (OR = 0.983, 95% CI = 0.979–0.988), ACAG (OR = 1.085, 95% CI = 1.057–1.113), creatine kinase–MB (OR = 1.007, 95% CI = 1.004–1.009), and the variables of shockable rhythm (OR = 0.187, 95% CI = 0.068–0.516) and cardiac origin of CA (OR = 0.482, 95% CI = 0.275–0.845) were retained in the final model (*p* < 0.05). The results are shown in Table 4 and Figure S1.

**TABLE 4:** Results of multivariate logistic regression for the entire population in the MIMIC‐IV database.

Parameter	*β*	SE	*p*	OR	95% CI
Intercept	−1.713	0.307	< 0.001	0.180	0.099–0.329
24‐h LCR	−0.017	0.002	< 0.001	0.983	0.979–0.988
ACAG	0.081	0.013	< 0.001	1.085	1.057–1.113
CK–MB	0.007	0.001	< 0.001	1.007	1.004–1.009
Defibrillate	−1.674	0.516	0.001	0.187	0.068–0.516
Cardiogenic	−0.730	0.286	0.011	0.482	0.275–0.845

*Note: β*: regression coefficient; CK–MB, creatine kinase–MB isoenzyme.

Abbreviations: 24‐h LCR, 24‐h lactate clearance rate; ACAG, albumin‐corrected anion gap; CI, confidence interval; OR, odds ratio; SE, standard error.

#### 3.4.3. Collinearity Analysis

Collinearity analysis showed that the VIF values for the five variables—24‐h LCR, ACAG, creatine kinase–MB isoenzyme, shockable rhythm, and cardiac origin of CA—were all less than 10, indicating no multicollinearity among these variables. Details are shown in Table 5.

**TABLE 5:** Collinearity analysis of variables in the model.

Parameter	*p*	VIF
24‐h LCR	< 0.001	1.010
ACAG	< 0.001	1.015
CK–MB	< 0.001	1.025
Defibrillate	0.001	1.008
Cardiogenic	0.011	1.029

*Note:* CK–MB: creatine kinase–MB isoenzyme.

Abbreviations: 24‐h LCR, 24‐h lactate clearance rate; ACAG, albumin‐corrected anion gap.

#### 3.4.4. Construction of a Temporary Mortality Risk Prediction Model on the Training Set

Patients from the MIMIC‐IV database were randomly divided into a training set and an internal validation set in a 7:3 ratio. The training set included 574 patients, with 378 in the survival group and 196 in the death group. The internal validation set included 247 patients, with 160 in the survival group and 87 in the death group. Based on the five variables selected above, a multivariate logistic regression model was constructed in the training set. The results are shown in Table S3.

#### 3.4.5. Evaluation of the Mortality Risk Prediction Model in the Internal Validation Set

In the internal validation set, a ROC curve was plotted to evaluate the model’s discriminative ability. The AUC was 0.775, with a 95% confidence interval (95% CI) of 0.714–0.836. When the risk threshold was set at the optimal cutoff value of 0.323 from the training set, the sensitivity for predicting death was 71.3%, and the specificity was 70.1%.

A calibration curve was plotted, and the H–L goodness‐of‐fit test was conducted to evaluate the model’s calibration. The H–L goodness‐of‐fit test yielded *X*^2^ = 11.28, *p* = 0.186 > 0.05, indicating a good fit for the model.

The Brier score was calculated to evaluate the model’s overall performance, resulting in a score of 0.1716 (0.25), indicating good predictive ability.

A clinical decision curve for the risk prediction model was plotted to evaluate its clinical applicability. The results demonstrated that at threshold probabilities ranging from 0.21 to 0.68, the net benefit of interventions guided by the risk prediction model in the internal validation cohort was superior to that of the default strategies (either intervening on all or no patients), indicating favorable clinical utility of the prediction model in the internal validation set. The contents are shown in Figure S2.

#### 3.4.6. Construction of the Final Mortality Risk Prediction Equation

**FIGURE 5:**
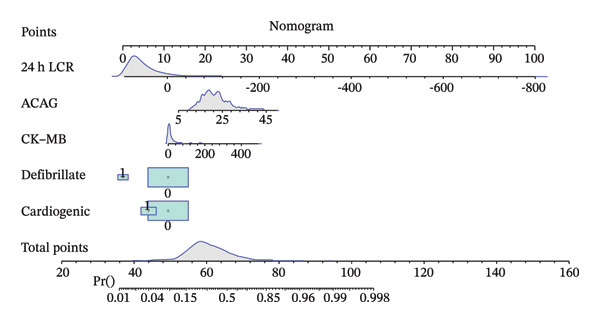
Nomogram for predicting the probability of short‐term (7‐day) mortality in cardiac arrest patients admitted to the ICU after ROSC.

Since the mortality risk prediction model for CA patients admitted to the ICU after ROSC performed well in the internal validation set, the final mortality risk prediction equation was constructed based on the variables selected in Table 4. The logistic regression model is as follows: *p*(*Y*) = 1/1 + *e*^ −[−1.713–0.017 × 24‐h lactate clearance rate + 0.081 × albumin‐corrected anion gap + 0.007 × creatine kinase–MB isoenzyme − 1.674 × shockable rhythm (=0 or 1) − 0.730 × cardiac origin of CA (=0 or 1)]. The nomogram is shown in Figure 5.

#### 3.4.7. External Validation and Evaluation of the Mortality Risk Prediction Model

A total of 273 CA patients who met the inclusion criteria were collected from the Second Affiliated Hospital of Soochow University as the external validation set. Among them, 140 were in the survival group and 133 were in the death group, with a mortality rate of 48.7%. The baseline characteristics of the MIMIC‐IV dataset and the external validation set were compared, and the results are shown in Table 6.

**TABLE 6:** Comparison of baseline characteristics between the MIMIC‐IV dataset and the external validation set.

Parameter	MIMIC‐IV dataset	External validation set	*χ* ^2^/*Z*	*p*
	(*n* = 821)	(*n* = 273)		
Age (years)	66.00 (55.00, 77.00)	65.00 (53.00, 77.00)	*Z* = −0.84	0.402
Weight (kg)	81.80 (69.47, 98.40)	67.00 (58.00, 75.00)	*Z* = −12.77	< 0.001
Height (cm)	170.00 (163.00, 178.00)	168.00 (162.00, 173.00)	*Z* = −4.68	< 0.001
Gender				
Male, *n* (%)	519 (63.22)	180 (65.93)	*χ* ^2^ = 0.66	0.418
Female, *n* (%)	302 (36.78)	93 (34.07)		
Complication				
Hypertension, *n* (%)	278 (33.86)	140 (51.28)	*χ* ^2^ = 26.34	< 0.001
Diabetes, *n* (%)	286 (34.84)	59 (21.61)	*χ* ^2^ = 16.59	< 0.001
Myocardial infarction, *n* (%)	170 (20.71)	30 (10.99)	*χ* ^2^ = 12.95	< 0.001
Renal dysfunction, *n* (%)	575 (70.04)	146 (53.48)	*χ* ^2^ = 24.99	< 0.001
Stroke, *n* (%)	77 (9.38)	45 (16.48)	*χ* ^2^ = 10.44	0.001
Chronic liver disease, *n* (%)	55 (6.70)	16 (5.88)	*χ* ^2^ = 0.22	0.636
Malignancy, *n* (%)	83 (10.11)	15 (5.49)	*χ* ^2^ = 5.35	0.021
Sepsis, *n* (%)	268 (32.64)	30 (10.99)	*χ* ^2^ = 48.47	< 0.001
24‐h LCR (%)	63.16 (38.46, 76.92)	50.00 (4.49, 68.10)	*Z* = −6.30	< 0.001
ACAG (mmol/L)	21.00 (17.50, 24.75)	23.89 (18.88, 30.86)	*Z* = −6.14	< 0.001
WBC (10^9/L)	14.20 (9.30, 20.00)	15.55 (11.28, 20.60)	*Z* = −2.53	0.011
RBC (10^12/L)	3.66 (3.00, 4.43)	4.12 (3.36, 4.70)	*Z* = −4.65	< 0.001
PLT (10^9/L)	193.00 (135.00, 260.00)	195.00 (139.50, 252.50)	*Z* = −0.20	0.843
Hb (g/dL)	10.90 (8.90, 13.00)	12.50 (10.10, 14.40)	*Z* = −5.46	< 0.001
PO_4_^3−^ (mg/dL)	4.40 (3.20, 5.80)	1.70 (1.05, 2.62)	*Z* = −19.56	< 0.001
RDW (%)	14.70 (13.60, 16.40)	45.20 (42.58, 48.82)	*Z* = −24.60	< 0.001
HCT (%)	34.00 (28.10, 40.80)	38.00 (31.00, 43.00)	*Z* = −4.54	< 0.001
Na^+^ (mmol/L)	139.00 (135.00, 142.00)	142.10 (139.20, 145.60)	*Z* = −9.36	< 0.001
K^+^ (mmol/L)	4.30 (3.80, 4.90)	3.85 (3.35, 4.41)	*Z* = −6.55	< 0.001
Cl^−^ (mmol/L)	104.00 (99.00, 108.00)	103.30 (98.97, 107.30)	*Z* = −1.22	0.222
Ca^2+^ (mmol/L)	1.10 (1.03, 1.17)	1.97 (1.83, 2.08)	*Z* = −24.45	< 0.001
GLU (mg/dL)	177.00 (129.00, 253.00)	207.00 (145.80, 282.60)	*Z* = −2.73	0.006
PH	7.28 (7.18, 7.36)	7.31 (7.20, 7.42)	*Z* = −3.22	0.001
PCO_2_ (mmHg)	43.00 (36.00, 52.00)	40.50 (32.00, 52.00)	*Z* = −2.45	0.014
PO_2_ (mmHg)	91.00 (50.00, 200.00)	116.00 (80.00, 207.00)	*Z* = −4.55	< 0.001
PT (s)	15.10 (12.90, 19.70)	16.10 (14.00, 19.70)	*Z* = −2.93	0.003
INR	1.40 (1.20, 1.80)	1.34 (1.11, 1.69)	*Z* = −2.06	0.039
TBIL (mg/dL)	0.60 (0.40, 1.20)	0.85 (0.52, 1.20)	*Z* = −3.58	< 0.001
ALT (U/L)	71.00 (29.00, 209.00)	104.00 (52.00, 270.50)	*Z* = −4.08	< 0.001
AST (U/L)	113.00 (48.00, 347.00)	239.00 (82.50, 614.00)	*Z* = −5.62	< 0.001
BUN (mg/dL)	24.00 (17.00, 39.00)	22.12 (17.08, 32.76)	*Z* = −2.22	0.026
Cr (mg/dL)	1.40 (1.00, 2.20)	1.26 (0.88, 1.75)	*Z* = −2.85	0.004
CK (U/L)	346.00 (124.00, 1041.00)	477.50 (181.00, 1334.75)	*Z* = −3.15	0.002
CK–MB (ng/mL)	9.00 (4.00, 28.00)	19.00 (8.00, 64.00)	*Z* = −6.41	< 0.001
cTnT (ng/mL)	0.22 (0.07, 0.75)	0.23 (0.07, 0.91)	*Z* = −0.62	0.535
Heart rate (bpm)	90.00 (77.00, 108.00)	101.50 (88.00, 117.00)	*Z* = −5.74	< 0.001
Systolic BP (mmHg)	115.00 (99.00, 132.00)	116.00 (98.00, 136.00)	*Z* = −0.01	0.992
Diastolic BP (mmHg)	62.00 (52.00, 73.00)	70.00 (57.75, 85.00)	*Z* = −5.82	< 0.001
Mean arterial pressure (mmHg)	79.00 (66.00, 93.00)	87.00 (71.00, 101.00)	*Z* = −4.36	< 0.001
Respiratory rate (bpm)	20.00 (16.00, 25.00)	16.00 (15.00, 20.00)	*Z* = −7.58	< 0.001
Oxygen saturation (%)	99.00 (95.00, 100.00)	100.00 (96.00, 100.00)	*Z* = −2.65	0.008
Temperature (°C)	36.78 (36.39, 37.17)	35.70 (34.60, 36.60)	*Z* = −11.41	< 0.001
Cardiogenic, *n* (%)	104 (12.67)	92 (33.70)	*χ* ^2^ = 61.63	< 0.001
Emergency admission, *n* (%)	136 (16.57)	229 (83.88)	*χ* ^2^ = 417.60	< 0.001
Defibrillate, *n* (%)	53 (6.46)	40 (14.65)	*χ* ^2^ = 17.70	< 0.001
Vasoactive agent				
Dopamine, *n* (%)	94 (11.45)	61 (22.34)	*χ* ^2^ = 20.00	< 0.001
Epinephrine, *n* (%)	185 (22.53)	31 (11.36)	*χ* ^2^ = 16.16	< 0.001
Norepinephrine, *n* (%)	514 (62.61)	221 (80.95)	*χ* ^2^ = 31.28	< 0.001
Renal replacement therapy, *n* (%)	162 (19.73)	61 (22.43)	*χ* ^2^ = 0.91	0.339
Invasive mechanical ventilation, *n* (%)	626 (76.25)	263 (97.05)	*χ* ^2^ = 58.24	< 0.001
SOFA score	9.00 (7.00, 12.00)	11.00 (9.00, 13.00)	*Z* = −5.57	< 0.001
SAPS II score	50.00 (39.00, 61.00)	45.00 (40.00, 49.00)	*Z* = −5.95	< 0.001
GCS score	15.00 (14.00, 15.00)	3.00 (3.00, 5.00)	*Z* = −20.77	< 0.001

*Note:* PTL: platelet count; HB: hemoglobin; PO_4_^3-^: phosphate; HCT: hematocrit; Na^+^: sodium ion; K^+^: potassium ion; Cl^−^: chloride ion; Ca^2+^: calcium ion; GLU: blood glucose; pH: acidity/alkalinity level; PCO_2_: arterial partial pressure of carbon dioxide; PO_2_: arterial partial pressure of oxygen; TBIL: total bilirubin; Cr: creatinine; CK–MB: creatine kinase–MB isoenzyme.

Abbreviations: 24‐h LCR, 24‐h lactate clearance rate; ACAG, albumin‐corrected anion gap; ALT, alanine aminotransferase; AST, aspartate aminotransferase; BUN, blood urea nitrogen; CK, creatine kinase; cTnT, cardiac troponin T; INR, International Normalized Ratio; PT, prothrombin time; RBC, red blood cell count; RDW, red cell distribution width; WBC, white blood cell count.

In the external validation set, a ROC curve was plotted to evaluate the model’s discriminative ability. The AUC was 0.743, with a 95% 95% CI of 0.685–0.801. When the risk threshold was set at the optimal cutoff value of 0.322 from the MIMIC‐IV dataset, the sensitivity for predicting death was 55.7%, and the specificity was 75.9%.

A calibration curve was plotted, and the H–L goodness‐of‐fit test was conducted to evaluate the model’s calibration. The H–L goodness‐of‐fit test yielded *X*^2^ = 3.648, *p* = 0.887 > 0.05, indicating a good fit for the model.

The Brier score was calculated to evaluate the model’s overall performance, resulting in a score of 0.1885 (0.25), indicating good predictive ability.

**FIGURE 6:**
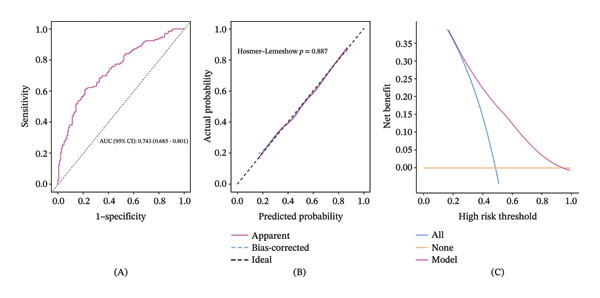
Evaluation of the mortality risk prediction model for cardiac arrest patients in the external validation set: (A) ROC curve in the external validation set; (B) calibration curve in the external validation set; and (C) clinical decision curve in the external validation set.

A clinical decision curve for the risk prediction model was plotted to evaluate its clinical applicability. The results demonstrated that at threshold probabilities ranging from 0.19 to 0.58, the net benefit of interventions guided by the risk prediction model in the external validation cohort was superior to that of the default strategies (either intervening on all or no patients), indicating favorable clinical utility of the prediction model in the external validation set. The contents are shown in Figure 6 and Table S4.

#### 3.4.8. Sensitivity Analysis: Complete‐Case Analysis

To evaluate the potential impact of multiple imputations on our model estimates, we conducted a complete‐case analysis using patients with no missing data for any of the five final predictors and the outcome. Among the 821 patients in the MIMIC‐IV derivation cohort, 517 (63%) had complete data for all five predictors and the outcome; the statistical interpolation data set has no obvious difference (Table S5). The AUC of the complete‐case model was 0.756 (95% CI 0.712–0.800), closely aligning with the AUC of 0.775 from the imputed model (Figure S3). These findings indicate that the imputation procedure did not materially influence the model’s coefficients or discriminatory performance, supporting the robustness of our conclusions.

## 4. Discussion

CA is one of the major global public health issues, with the number of new cases in China increasing year by year [20]. Although the success rate of ROSC has improved recently, the overall prognosis of most CA patients remains poor. The main value of this study is not simply that lactate clearance or ACAG was statistically associated with mortality. Rather, the study addresses a narrower and clinically relevant question: Among patients with CA who have survived the first 24 h after ROSC and have routine post‐ROSC laboratory data available, can a small set of readily obtainable variables help stratify short‐term mortality risk? In this context, 24‐h LCR and ACAG provide complementary information on dynamic metabolic recovery and residual acid‐base derangement, while CK–MB, shockable rhythm, and cardiac origin of arrest add clinically interpretable information on myocardial injury and arrest phenotype.

The proposed model should therefore be understood as a post‐24 h risk stratification tool, not as a model for immediate decision‐making during resuscitation or during the first hours after ROSC. Patients who die within the first 24 h often have incomplete serial lactate measurements, making 24‐h LCR impossible to calculate in practice. More importantly, death in this time window may be driven predominantly by pre‐ROSC characteristics and resuscitation‐process variables, such as arrest location, no‐flow and low‐flow duration, CPR quality, initial rhythm, and immediate hemodynamic collapse. By contrast, our model was designed to evaluate whether post‐ROSC metabolic and clinical features could identify patients who remain at high risk after they have survived to the 24‐h assessment point. These are related but different prognostic questions and likely require different predictors and study designs.

From a clinical perspective, the model may be useful because all five final predictors are routinely available in most intensive care settings and do not require specialized testing. A high predicted risk should not be used in isolation to limit treatment or determine withdrawal of life‐sustaining therapy. Instead, it may prompt clinicians to reassess potentially reversible metabolic acidosis, persistent lactate elevation, myocardial injury, and hemodynamic instability; to increase the intensity of monitoring; to involve multidisciplinary teams earlier; and to identify patients who may be suitable for enrollment in interventional or prognostic studies. In this sense, the model is intended to support structured risk communication and bedside prioritization rather than replace comprehensive clinical judgment.

The interpretation of LCR requires caution. Lactate is increasingly understood as a marker of systemic metabolic stress and energy‐state dysregulation rather than a direct measure of tissue perfusion or oxygenation alone. Previous studies have shown that lactate clearance is associated with outcomes in critically ill patients, including cardiogenic shock and other severe acute illnesses [21–23]. In post‐CA populations, lactate dynamics have also been associated with survival, although the optimal time point for assessment has varied across studies [24, 25]. In our study, 24‐h LCR performed better than 6‐h and 12‐h LCR when combined with ACAG, suggesting that later metabolic recovery may carry additional prognostic information in patients who survive to this assessment point.

ACAG captures a related but distinct aspect of post‐arrest metabolic derangement. While LCR reflects the direction of metabolic recovery over time, ACAG reflects residual albumin‐corrected acid‐base burden. This is clinically relevant because hypoalbuminemia may mask the severity of metabolic acidosis when the uncorrected AG is used. Prior studies have reported that ACAG is associated with prognosis in cardiopulmonary arrest and cardiovascular critical illness [26, 27]. Therefore, combining LCR and ACAG may provide a broader metabolic assessment than either marker alone: LCR reflects dynamic improvement or deterioration, whereas ACAG reflects the remaining acid‐base disturbance at baseline.

The other variables retained in the model also have plausible clinical meaning. CK–MB reflects myocardial injury, which is common after CA and may contribute to post‐ROSC circulatory instability; previous studies have linked CK–MB elevation with adverse outcomes in cardiovascular emergencies [28]. A shockable rhythm is generally associated with better prognosis because it is more amenable to prompt defibrillation and may indicate a more reversible arrest mechanism [29–31]. Similarly, cardiac origin of arrest may be associated with a more identifiable and potentially treatable cause, such as acute coronary syndrome, compared with noncardiac causes such as severe infection, respiratory failure, or metabolic collapse [32, 33]. The final model therefore combines metabolic recovery, acid‐base status, myocardial injury, and arrest phenotype, rather than relying on a single biomarker.

Compared with previous studies, our work extends rather than replaces the existing evidence. Earlier studies have mainly evaluated lactate, lactate clearance, or ACAG as individual prognostic markers after CA or in related critical illness settings. The present study integrates these routinely available variables into a multivariable nomogram and evaluates discrimination, calibration, decision‐curve performance, and external validation. The resulting performance was moderate rather than definitive, but the model has the advantage of being simple, interpretable, and based on variables that are commonly available in ICU practice.

The external validation cohort differed from the MIMIC‐IV derivation cohort in several baseline characteristics and had a higher mortality rate. Such heterogeneity may reduce calibration, but it is also an important test of whether the model can preserve risk ranking across different clinical settings. Differences in patient selection, arrest location, severity, lactate measurement timing, and outcome definitions are common in post‐CA prognostic studies. In our study, the external AUC remained acceptable, suggesting that the model retained some discriminative ability despite case‐mix differences. However, this should not be interpreted as proof of universal transportability. Before clinical deployment in substantially different populations, local recalibration of the intercept or slope and further multicenter validation are necessary.

Although the prediction model in this study performed well in short‐term mortality risk assessment, it has the following limitations: Firstly, the MIMIC‐IV data mainly comes from a single center [34], which may affect the model’s generalizability. Although validation was performed, the validation set was small, and further multicenter studies are needed to validate the model’s generalization ability. Secondly, due to database limitations, factors that may affect prognosis, such as bystander CPR, resuscitation time, and brain natriuretic peptide levels, were not included. Additionally, the lack of 72 h neurological function assessment data limits the model’s ability to predict neurological outcomes. Thirdly, this study only focused on 7‐day mortality risk in the ICU and did not assess long‐term survival rates and functional outcomes. Future studies could further expand the analysis scope. Fourthly, the requirement for serial lactate measurements up to 24 h may have introduced survival bias by excluding patients who died early or did not have repeated blood draws. This limits the generalizability of our model to very early post‐arrest settings or hospitals with less frequent laboratory monitoring.

In addition to the above limitations, several further issues should be emphasized. Because the derivation cohort was identified using ICD codes in MIMIC‐IV, the exact location of CA could not be reliably distinguished for all patients. Therefore, in‐hospital and out‐of‐hospital CA patients could not be analyzed separately, which may have introduced clinical heterogeneity and affected calibration and generalizability. Future studies with prospectively collected data should validate and, if necessary, recalibrate the model separately in IHCA and OHCA populations. Moreover, the outcome of this study was 7‐day all‐cause mortality, and cause‐specific death could not be adjudicated. The database does not reliably distinguish cardiac death, neurological death, or death due to multiorgan failure, and formal neurological injury variables or neurological outcome assessments were unavailable. In clinical practice, assigning a single dominant cause of death after CA can also be difficult because PCAS often involves overlapping myocardial dysfunction, brain injury, systemic ischemia‐reperfusion injury, and multiorgan failure. Future studies should include larger multicenter cohorts, explicit IHCA/OHCA classification, earlier time points within the first 24 h, cause‐specific outcomes, and long‐term neurological endpoints.

In summary, this study proposes a simple and interpretable model for 7‐day mortality risk stratification among post‐CA patients who survive to the 24‐h assessment point. The model is not intended for immediate resuscitation‐phase decision‐making or for the first hours after ROSC, but it may help clinicians identify persistently high‐risk patients after the first 24 h following ROSC. Further prospective, multicenter studies are needed to validate the model in clearly defined IHCA and OHCA cohorts, evaluate earlier time‐point models, assess cause‐specific and neurological outcomes, and determine whether recalibrated versions improve clinical decision‐making.

## 5. Conclusion

1. Overall, 6‐h LCR, 12‐h LCR, 24‐h LCR, and ACAG can all predict short‐term (7‐day) mortality in the ICU, and the predictive performance of 24 h LCR combined with ACAG is higher than that of other time points combined with ACAG and also higher than the predictive performance of each indicator alone.
2. Overall, 24‐h LCR, ACAG, and CK–MB after ROSC; whether the patient has a shockable rhythm; and whether the CA is of cardiac origin are independent influencing factors for short‐term (7‐day) mortality in the ICU for CA patients after ROSC.
3. The short‐term (7‐day) mortality risk prediction model for CA patients after ROSC constructed in this study has good predictive performance and certain clinical reference value.

## Author Contributions

Dr Yani Gao, Sheng Bi, and Haoran Li contributed equally; Yan Xiao and Xuming Zhao are co‐correspondence authors; Yani Gao and Haoran Li made project development, data collection or management, data analysis, and manuscript writing/editing; Yan Xiao, Xuming Zhao, and Chaogang Wei made project development and data collection and management; Xiaojin Sun made data collection and management and manuscript writing/editing; Xuming Zhao and Sheng Bi made data analysis and manuscript writing and revision.

## Funding

This research was supported in part by the Project of National Natural Science Foundation no. 82372206, the Project of Jiangsu Provincial Health Commission H2023107, and the project of basic and clinical research on cardiac arrest in the Emergency and Critical Care Department of the Second Affiliated Hospital of Soochow University (XKTJ‐XK202408‐2).

## Ethics Statement

This study has been approved by the Ethics Review Committee of the Second Affiliated Hospital of Soochow University (Ethics number: JD‐HG‐2024‐102).

For the MIMIC‐IV database cohort, informed consent was waived because the database contains de‐identified, anonymized data from a publicly available source, and the requirement for informed consent was previously approved by the Institutional Review Boards of Beth Israel Deaconess Medical Center and the Massachusetts Institute of Technology. The author (Yani Gao) completed the Collaborative Institutional Training Initiative (CITI) program and obtained certification (certificate number: 62057797) to access and use the database.

For the external validation cohort from the Second Affiliated Hospital of Soochow University, written informed consent was obtained from all participants or their legally authorized representatives prior to enrollment. For patients who were unable to provide consent due to critical illness or unconsciousness at the time of admission, consent was obtained from their next of kin or legal guardians. All data were anonymized prior to analysis.

## Conflicts of Interest

The authors declare no conflicts of interest.

## Supporting Information

Additional supporting information can be found online in the Supporting Information section.

## Supporting information


**Supporting Information 1** Table S1: Variables deleted due to excessive missing values in the MIMIC‐IV database. Table S2: Univariate logistic regression results for the entire population in the MIMIC database. Table S3: Multivariate logistic regression results in the training set. Table S4: Model confusion matrix correlation parameters. Table S5: Comparison of baseline data in datasets after deletion of missing values. Figure S1: Forest plot of multivariate logistic regression analysis for the entire population in the MIMIC‐IV database. Figure S2: Evaluation of the mortality risk prediction model for cardiac arrest patients in the internal validation set: (A) ROC curve in the internal validation Set; (B) calibration curve in the internal validation Set; (C) clinical decision curve in the internal validation set. Figure S3: Evaluation of the mortality risk prediction model for cardiac arrest patients in the complete‐case analysis: (A) ROC curve in the internal validation set; (B) calibration curve in the internal validation set; (C) clinical decision curve in the internal validation set.

**Supporting Information 2** STROBE_checklist_cohort.

## Data Availability

The data supporting the results of this study are available from the corresponding author upon reasonable request. The datasets generated from other sources are available in the MIMIC repositories, https://mimic.physionet.org/.
