# Supplementary material for: Construction of a Mortality Prediction Model for Cardiac Arrest Patients Using Lactate Clearance Rate and Albumin‐Corrected Anion Gap
Source: Emerg Med Int. 2026 Aug 17;2026:3964486. doi: 10.1155/emmi/3964486 (PMC13478800; doi:10.1155/emmi/3964486)
Supplement: Supplementary file 1 [file EMMI-2026-3964486-s001.docx]

**Supplementary Information Files**

****Table S1 Variables Deleted Due to Excessive Missing Values in the MIMIC-IV Database****

| **Clinical Data** | **Missing Values (n)** | **Missing Rate (%)** |
| --- | --- | --- |
| C-Reactive Protein | 750 | 91.4 |
| Fibrinogen | 324 | 39.5 |
| D-Dimer | 799 | 97.3 |
| Triglycerides | 511 | 62.2 |
| Total Cholesterol | 709 | 86.4 |
| Uric Acid | 762 | 92.8 |
| N-Terminal ProBNP | 711 | 86.6 |

****Table S2 Univariate Logistic Regression Results for the Entire Population in the MIMIC Database****

| **Parameter** | ***β*** | **OR** | **95%CI** | ***P*** |
| --- | --- | --- | --- | --- |
| **Age** | -0.002 | 1 | 0.99 ~ 1.01 | 0.644 |
| **Weight** | 0.002 | 1 | 1.00 ~ 1.01 | 0.377 |
| **Height** | 0.001 | 1 | 0.99 ~ 1.01 | 0.902 |
| **Gender (Male)** | -0.159 | 0.85 | 0.63 ~ 1.15 | 0.294 |
| **Comorbid Hypertension** | -0.044 | 0.96 | 0.71 ~ 1.30 | 0.777 |
| **Comorbid Diabetes** | -0.134 | 0.87 | 0.65 ~ 1.19 | 0.390 |
| **Comorbid Myocardial Infarction** | 0.111 | 1.12 | 0.79 ~ 1.59 | 0.538 |
| **Comorbid Renal Dysfunction** | -0.031 | 1.19 | 0.87 ~ 1.64 | 0.276 |
| **Comorbid Stroke** | -0.165 | 0.85 | 0.51 ~ 1.41 | 0.522 |
| **Comorbid Chronic Liver Disease** | -0.084 | 0.92 | 0.51 ~ 1.65 | 0.778 |
| **Comorbid Malignancy** | 0.420 | 1.52 | 0.96 ~ 2.41 | 0.073 |
| **Comorbid Sepsis** | -0.183 | 0.83 | 0.61 ~ 1.14 | 0.248 |
| 24h LCR ^a^ | -0.014 | 0.99 | 0.98 ~ 0.99 | <0.001 |
| ACAG ^b^ | 0.065 | 1.07 | 1.04 ~ 1.09 | <0.001 |
| WBC ^c^ | 0.022 | 1.02 | 1.01 ~ 1.04 | 0.015 |
| RBC ^d^ | 0.187 | 1.21 | 1.04 ~ 1.40 | 0.016 |
| PLT ^e^ | 0.002 | 1.01 | 1.01 ~ 1.01 | 0.018 |
| Hb ^f^ | 0.076 | 1.08 | 1.02 ~ 1.14 | 0.004 |
| PO_4_^3-g^ | 0.112 | 1.12 | 1.05 ~ 1.19 | <0.001 |
| RDW ^h^ | 0.041 | 1.04 | 0.99 ~ 1.10 | 0.147 |
| HCT ^i^ | 0.028 | 1.03 | 1.01 ~ 1.05 | 0.001 |
| Na^+ j^ | 0.009 | 1.01 | 0.98 ~ 1.03 | 0.473 |
| K^+ k^ | -0.020 | 0.98 | 0.85 ~ 1.14 | 0.792 |
| Cl^- l^ | -0.014 | 0.99 | 0.97 ~ 1.01 | 0.178 |
| Ca^2+ m^ | -0.371 | 0.69 | 0.26 ~ 1.81 | 0.450 |
| GLU ^n^ | 0.001 | 1.01 | 1.01 ~ 1.01 | 0.023 |
| PH ^o^ | -2.078 | 0.13 | 0.04 ~ 0.38 | <0.001 |
| PCO_2_ ^p^ | 0.002 | 1 | 0.99 ~ 1.01 | 0.73 |
| PO_2_ ^q^ | -0.002 | 0.99 | 0.99 ~ 0.99 | <0.001 |
| PT ^r^ | 0.011 | 1.01 | 1.01 ~ 1.02 | 0.044 |
| INR ^s^ | 0.112 | 1.12 | 0.99 ~ 1.26 | 0.071 |
| TBIL ^t^ | 0.024 | 1.02 | 0.98 ~ 1.07 | 0.286 |
| ALT ^u^ | 0.001 | 1.01 | 1.01 ~ 1.01 | 0.003 |
| AST ^v^ | 0.001 | 1.01 | 1.01 ~ 1.01 | 0.011 |
| BUN ^w^ | 0.003 | 1 | 1.00 ~ 1.01 | 0.388 |
| Cr ^x^ | 0.001 | 1 | 0.93 ~ 1.08 | 0.974 |
| CK ^y^ | 0.000 | 1 | 1.00 ~ 1.00 | 0.678 |
| CK-MB ^z^ | 0.006 | 1.01 | 1.01 ~ 1.01 | <0.001 |
| cTnT ^A^ | 0.041 | 1.04 | 0.99 ~ 1.10 | 0.124 |
| Heart Rate | 0.002 | 1 | 1.00 ~ 1.01 | 0.496 |
| **Systolic Blood Pressure** | -0.001 | 1 | 0.99 ~ 1.00 | 0.786 |
| **Diastolic Blood Pressure** | 0.003 | 1 | 0.99 ~ 1.01 | 0.535 |
| **Mean Arterial Pressure** | 0.002 | 1 | 1.00 ~ 1.00 | 0.286 |
| **Respiratory Rate** | 0.018 | 1.02 | 1.00 ~ 1.04 | 0.092 |
| **Oxygen Saturation** | -0.006 | 0.99 | 0.97 ~ 1.02 | 0.581 |
| **Temperature** | -0.016 | 0.98 | 0.96 ~ 1.01 | 0.182 |
| **Cardiogenic Cardiac Arrest** | -0.516 | 0.6 | 0.37 ~ 0.95 | 0.031 |
| **Emergency Admission** | -0.195 | 0.82 | 0.55 ~ 1.22 | 0.336 |
| **Defibrillate** | -1.695 | 0.18 | 0.07 ~ 0.47 | <0.001 |
| **Use of Dopamine** | 0.187 | 1.21 | 0.77 ~ 1.88 | 0.407 |
| **Use of Epinephrine** | 0.190 | 1.21 | 0.86 ~ 1.70 | 0.274 |
| **Use of Norepinephrine** | 0.468 | 1.6 | 1.18 ~ 2.17 | 0.003 |
| **Renal Replacement Therapy** | 0.304 | 1.36 | 0.95 ~ 1.93 | 0.092 |
| **Invasive Mechanical Ventilation** | 0.313 | 1.37 | 0.96 ~ 1.94 | 0.079 |
| **SOFA Score** | 0.094 | 1.1 | 1.06 ~ 1.14 | <0.001 |
| **SAPS II Score** | 0.002 | 1 | 0.99 ~ 1.01 | 0.648 |
| **GCS Score** | 0.033 | 1.03 | 1.00~ 1.07 | 0.074 |

**Note:** *β*: Regression coefficient; OR: Odds ratio; CI: Confidence interval;
a: 24h LCR: 24-hour lactate clearance rate; b: ACAG: Albumin-corrected anion gap; c: WBC: White blood cell count; d: RBC: Red blood cell count; e: PTL: Platelet count; f: HB: Hemoglobin; g: PO₄³⁻: Phosphate; h: RDW: Red cell distribution width; i: HCT: Hematocrit; j: Na⁺: Sodium ion; k: K⁺: Potassium ion; l: Cl⁻: Chloride ion; m: Ca²⁺: Calcium ion; n: GLU: Blood glucose; o: pH: Acidity/alkalinity level; p: PCO₂: Arterial partial pressure of carbon dioxide; q: PO₂: Arterial partial pressure of oxygen; r: PT: Prothrombin time; s: INR: International normalized ratio; t: TBIL: Total bilirubin; u: ALT: Alanine aminotransferase; v: AST: Aspartate aminotransferase; w: BUN: Blood urea nitrogen; x: Cr: Creatinine; y: CK: Creatine kinase; z: CK-MB: Creatine kinase-MB isoenzyme; A: cTnT: Cardiac troponin T.

****Table S3 Multivariate Logistic Regression Results in the Training Set****

| **Parameter** | ***β*** | **S.E** | ***P*** | **OR** | **95%CI** |
| --- | --- | --- | --- | --- | --- |
| Intercept | -1.726 | 0.369 | <0.001 | 0.178 | 0.086~0.366 |
| 24h LCR ^a^ | -0.015 | 0.003 | <0.001 | 0.985 | 0.980~0.991 |
| ACAG ^b^ | 0.077 | 0.016 | <0.001 | 1.080 | 1.046~1.114 |
| CK-MB ^c^ | 0.007 | 0.001 | <0.001 | 1.007 | 1.004~1.010 |
| defibrillate | -1.411 | 0.582 | 0.015 | 0.244 | 0.078~0.763 |
| Cardiogenic | -0.953 | 0.358 | 0.008 | 0.386 | 0.191~0.778 |

**Note:** *β*: Regression coefficient; SE: Standard error; OR: Odds ratio; CI: Confidence interval;

a: 24h LCR: 24-hour lactate clearance rate; b: ACAG: Albumin-corrected anion gap; c: CK-MB: Creatine kinase-MB isoenzyme.

****Table S4 Model Confusion Matrix Correlation Parameters****

| Group | AUC (95%CI) | Accuracy (95%CI) | Sensitivity (95%CI) | Specificity (95%CI) | PPV (95%CI) | NPV (95%CI) | cut off |
| --- | --- | --- | --- | --- | --- | --- | --- |
| Internal Validation Set | 0.775 (0.714-0.836) | 0.733 (0.673-0.787) | 0.713 (0.622 - 0.804) | 0.701 (0.632 - 0.770) | 0.596 (0.504 - 0.688) | 0.841 (0.780 - 0.902) | 0.323 |
| External Validation Set | 0.743 (0.685-0.801) | 0.740 (0.684-0.791) | 0.557 (0.474 - 0.640) | 0.759 (0.704 - 0.813) | 0.823 (0.747 - 0.899) | 0.695 (0.627 - 0.763) | 0.322 |

****Table S5 Comparison of Baseline Data in datasets after deletion of missing values****

| **Variables** | **After Imputation (n = 821)** | **Complete data before interpolation (n = 517)** | ***χ² /Z*** | **P** |
| --- | --- | --- | --- | --- |
|  |  |  |  |  |
| X24h LCR, Mean ± SD | 46.78 ± 63.21 | 46.95 ± 69.10 | Z=-0.05 | 0.962 |
| ACAG, Mean ± SD | 21.76 ± 6.27 | 21.91 ± 6.16 | Z=-0.41 | 0.680 |
| CKMB, Mean ± SD | 34.62 ± 70.76 | 33.55 ± 69.62 | Z=0.27 | 0.786 |
| Cardiogenic , n(%) |  |  | χ²=1.78 | 0.182 |
|  | 104 (12.67) | 59 (11.4) |  |  |
| Defibrillate, n(%) |  |  | χ²=0.01 | 0.930 |
|  | 53 (6.46) | 34 (6.58) |  |  |

### ****Figure S1 Forest plot of multivariate logistic regression analysis for the entire population in the MIMIC-IV database****

### **Figure S2 Evaluation of the Mortality Risk Prediction Model for Cardiac Arrest Patients in the Internal Validation Set : (A) ROC Curve in the Internal Validation Set; (B) Calibration Curve in the Internal Validation Set; (C) Clinical Decision Curve in the Internal Validation Set**

A

B

C

### **Figure S3 Evaluation of the Mortality Risk Prediction Model for Cardiac Arrest Patients in the complete-case analysis : (A) ROC Curve in the Internal Validation Set; (B) Calibration Curve in the Internal Validation Set; (C) Clinical Decision Curve in the Internal Validation Set**

**Calculation of Study Indicators**

This study was an observational study. The sample size was calculated based on the estimation of population proportion. Initially, a total of 1,968 patients were screened, with a mortality rate of 32%. Substituting π=32% into the formula, with a permissible error δ set at 0.05, α=0.05, and u_α/2_=u_0.05/2_=1.96, the calculated sample size was n=1.96²×0.32×(1-0.32)/0.05²=334. Considering the high missing rate of predictor variables (58%), the adjusted sample size was calculated using the formula n_adjusted=n/(1-missing rate) yielding 334/(1-0.58)=795. The modeling cohort in this study ultimately included 821 patients from the MIMIC-IV database, which meets the sample size requirement.

$$\text{n}=\frac{\text{u}^{2}_{\alpha/2}\pi(1-\pi)}{\delta^{2}}$$

The calculation formulas for the research indicators are as follows:

Lactate clearance rate (LCR) calculation formula: LCR (%) = (initial lactate - lactate at corresponding time) / initial lactate × 100%; Negative LCR values (indicating a rise in lactate) were permitted and included in analyses. No winsorization, capping, or exclusion was applied for low baseline lactate values; all available data were used as calculated.

Anion gap (AG) calculation formula: AG (mmol/L) = (sodium + potassium) - (chloride + bicarbonate);

Albumin-corrected anion gap (ACAG) calculation formula: ACAG (mmol/L) = [4.4 - albumin (g/dL)] × 2.5 + AG.
